# *De Novo* Transcriptome Assembly in Chili Pepper (*Capsicum frutescens*) to Identify Genes Involved in the Biosynthesis of Capsaicinoids

**DOI:** 10.1371/journal.pone.0048156

**Published:** 2013-01-22

**Authors:** Shaoqun Liu, Wanshun Li, Yimin Wu, Changming Chen, Jianjun Lei

**Affiliations:** College of Horticulture, South China Agricultural University, Guangzhou, China; Kyushu Institute of Technology, Japan

## Abstract

The capsaicinoids are a group of compounds produced by chili pepper fruits and are used widely in many fields, especially in medical purposes. The capsaicinoid biosynthetic pathway has not yet been established clearly. To understand more knowledge in biosynthesis of capsaicinoids, we applied RNA-seq for the mixture of placenta and pericarp of pungent pepper (*Capsicum frutescens* L.). We have assessed the effect of various assembly parameters using different assembly software, and obtained one of the best strategies for *de novo* assembly of transcriptome data. We obtained a total 54,045 high-quality unigenes (transcripts) using Trinity software. About 92.65% of unigenes showed similarity to the public protein sequences, genome of potato and tomato and pepper (*C. annuum*) ESTs databases. Our results predicted 3 new structural genes (DHAD, TD, PAT), which filled gaps of the capsaicinoid biosynthetic pathway predicted by Mazourek, and revealed new candidate genes involved in capsaicinoid biosynthesis based on KEGG (Kyoto Encyclopedia of Genes and Genomes) analysis. A significant number of SSR (Simple Sequence Repeat) and SNP (Single Nucleotide Polymorphism) markers were predicted in *C. frutescens* and *C. annuum* sequences, which will be helpful in the identification of polymorphisms within chili pepper populations. These data will provide new insights to the pathway of capsaicinoid biosynthesis and subsequent research of chili peppers. In addition, our strategy of *de novo* transcriptome assembly is applicable to a wide range of similar studies.

## Introduction

Pepper (*Capsicum* spp.) is one of the most economically and agriculturally important vegetable crops on the globe, with high consumption of fresh or processed products. One of the important attributes of pepper is pungency, which is caused by alkaloid compounds that are synthesized and accumulated in pepper fruit [1]. These compounds have been widely studied and used in the food, medical, and pharmaceutical industries [2]–[8]. In fact, in clinical evaluations, it has been validated that the capsaicinoids possess antioxidant, anticancer, antiarthritic, and analgesic properties [9].

Capsaicinoids have been studied since the beginning of the 1800s, and the general capsaicinoid biosynthetic pathway was established at the end of the 1960s [1], [10], [11]. Their results indicated that capsaicinoids are synthesized by the condensation of vanillylamine, derived from L-phenylalanine, with a branched-chain fatty acid, derived from either valine or leucine [1], [10], [12]. The sequential synthesis of phenylalanine, cinnamic, p-coumaric, caffeic and ferulic acids, and then the formation of vanillin and vanillylamine were involved in the proposed biosynthetic phenylpropanoid pathway at that time [1]. Some of these enzymes, such as phenylalanine ammonia lyase (PAL), cinnamate 4-hydroxylase (C4H), coumarate 3-hydroxylase (C3H) and caffeic acid O-methyltransferase (COMT) related with phenylpropanoid-mediated capsaicinoid biosynthesis have been described in previous papers [13], [14], [15]. Stewart et al. [16] identified other enzymes in the phenylpropanoid pathway that catalyze capsaicinoid formation, such as 4-coumaroyl-CoA ligase (4CL), hydroxycinnamoyl transferase (HCT), caffeoyl- CoA O-methyltransferase (CCoAOMT; instead of COMT) and hydroxycinnamoyl-CoA hydratase/lyase (HCHL). Recently, Mazourek et al. [17] proposed an innovative branched-chain fatty acid biosynthetic pathway, proposing that, in addition to isobutyryl-CoA, some other intermediaries like acetyl-CoA, isovaleryl-CoA, anteisovaleryl-CoA and propinyl-CoA could be used as substrates for capsaicinoid biosynthesis [11], [17]. In addition, they implemented a comprehensive model of capsaicinoid biosynthesis and made it publicly available within the SolCyc database at the SOL Genomics Network (http://www.sgn.cornell.edu). As a preliminary test of this model, and to build its value as a resource, targeted transcripts were cloned as candidates for nearly all of the structural genes for capsaicinoid biosynthesis [17]. However, there are still some unknown genes involved in this process to be identified, such as Dihydroxyacid dehydratase (DHAD), Thr deaminase (TD) and Prephenate aminotransferase (PAT).

The molecular mechanism of biosynthesis of capsaicinoids has been a matter of research for decades and many genes were studied, such as phenylalanine ammonia-lyase (PAL), cinnamic acid 4-hydroxylase (C4H), cafieic acid O-methyltransferase (COMT), a putative aminotransferase (pAMT) and a *β*-keto-acyl- [acyl-carrier-protein] synthase (KAS), fatty acid synthase (FAS) genes *Acl1*, *FatA*, acyltransferase gene (*At3*), acyl-transferase gene(*Catf-1*) [18]–[23]. These studies notwithstanding, the mechanism of the capsaicinoid biosynthetic pathway is not well understood.

In this study, a number of candidate genes involved in the biosynthesis of capsaicinoids were identified; the sequence of Dihydroxyacid dehydratase (DHAD), Thr deaminase (TD) and Prephenate aminotransferase (PAT) were predicted; and thousands of molecular markers were detected.

## Materials and Methods

### Plant materials

Xiaomila (*Capsicum frutescens* Linn.) is a kind of pungent pepper, mainly distributed in the southern Yunnan Province of China. It is perennial shrub-like plant, and it is the only wild pepper resource in China [24]. Under long-term pressures of natural selection, Xiaomila now possesses some characteristics such as high temperature and humidity tolerance, disease resistance, and growth in barren and low-light conditions. In addition, Xiaomila produces abundant fruit with fragrant pulp. In our research, the experimental material was cultivated in the experimental farm of South China Agriculture University.

Placenta and pericarp were harvested from 20 to 30 DAF (Days After Flowering) and the flower buds at different development stages were collected and blended. These tissues were collected, marked, frozen immediately in liquid nitrogen, and stored at −80°C for future RNA extraction.

### RNA-Seq library preparation and sequencing

Total RNA was extracted from different tissues using TRIzol reagents (Invitrogen, USA) according to the manufacturer's instruction. Each RNA sample was subjected to DNase digestion (Takara, Dalian, China) to remove any remaining DNA, and pooled equally. At least 20 µg of total RNA (≥400 ng/µL) was used to prepare a standard cDNA library for sequence analysis via Illumina HiSeq™ 2000 (commercial service) at the Beijing Genomics Institute (Shenzhen, China).

### RNA-Seq data filter

Each fragment used pair-end sequencing (PE) from a 200 bp insert library, and the length of each sequence read is 90 bp. We removed the low-quality reads with these criteria: 1) the read contain the sequencing adaptor was removed and 2) the low-quality nucleotide was trimmed using a customer Perl script (CONDETRI: http://code.google.com/p/condetri) with these parameters (-hq = 20 -lq = 10 -frac = 0.8 -lfrac = 0.1 -minlen = 50 -mh = 5 -ml = 5 -sc = 64 and other default paratemers) [25]. All reads were deposited in the National Center for Biotechnology Information (NCBI) and can be accessed in the Short Read Archive (SRA) under accession number: SRA048361.1.

### Assessment of assembly program and parameters

In our research, we used Trinity and Velvet-oases (Velvet followed by Oases) for *de novo* assembly of these reads to generate a non-redundant set of transcripts. We validated the publicly available programs Trinity (trinityrnaseq_r2012-05-18; http://trinityrnaseq.sourceforge.net/) and Velvet-oases (Velvet: version 1.2.07, http://www.ebi.ac.uk/~zerbino/velvet/ and Oases: version 0.2.08, http://www.ebi.ac.uk/~zerbino/oases/). They have been developed for transcriptome assembly of short reads using de Bruijn graph algorithm [26], [27]. Various k-mer were also optimized for the best result. Only one k-mer length (25-mer) was chosen in Trinity, using the follow parameters: group_pairs_distance = 250, path_reinforcement_distance = 70, min_glue = 2, min_kmer_cov = 2 and other default parameters. In Velvet-oases, we chose 7 different k-mers (21, 25, 29, 31, 37, 41 and 47) and other default parameters.

To evaluate the accuracy of the assembled sequences (transcripts), we realigned all the usable sequencing reads onto the transcripts using SOAPaligner (Release 2.21, 08-13-2009), allowing up to 3 base mismatches and a minimum length of 45 bp. We calculated the reads coverage of each transcript, except the 45 bp at both end of the transcript. If the reads coverage (the depth at least one read) is over 90% of the transcript, this transcript was defined positive.

### Analysis of Transcript Assembly

We used optimal programs and parameters in our study. After Trinity assembly, we used the TGI Clustering Tool (TGICL; [28]) followed by Phrap assembler for obtaining distinct sequences (unigenes). The following parameters were used to ensure quality of assembly: a minimum of 95% identity, a minimum of 35 overlapping bases, a minimum of 35 scores and a maximum of 20 unmatched overhanging bases at sequence ends.

### Annotation and predicted CDS

We annotated the unigenes in three ways. Firstly, the unigenes were aligned to three public protein databases (Nr, Swiss-Prot and KEGG) by blastx, and the cut-off E-value was 1.0e−5. Then, unigenes were annotated by the potato genome [29] (http://solgenomics.net/organism/Solanum_tuberosum/genome) and tomato genome [30] (http://solgenomics.net/organism/Solanum_lycopersicum/genome) and their whole proteome using blat and blastx, respectively. The cut-off E-value was 1.0e−5. The last, using blastn and tblastx with the cut-off E-value of 1.0e−10, an analysis of homologous was carried out against public pepper (C. annuum) ESTs database, which comprises the follow data: two *C. annuum* varieties, Serrano Tampiqueño 74 and Sonora Anaheim mixed tissues [31] (http://www.bioingenios.ira.cinvestav.mx:81/Joomla/), Bukang (*C. annuum* L.) mature fruit cDNA library (NCBI dbEST ID: 23667) [32] and TF68 (*C. annuum* L) mature fruit (GEO accession No. GSE29215) [33].

We predicted the CDS (Coding sequences) using blastx and ESTscan [34]. We searched unigene sequences in protein databases using blastx (e-value<1.0e−5) in the following order: Nr, SwissProt and KEGG. Unigenes with sequences having matches in one database were not searched further. We selected CDS from unigene sequences based on the blastx result, and the CDS were also used to train ESTScan. Unigenes not uncovered in blastx were predicted by ESTScan. The shortest CDS were at least 100 bp.

### Putative Molecular Markers

The MISA Perl script (http://pgrc.ipk-gatersleben.de/misa/) was used for identification of SSRs (Simple Sequence Repeat). Mono-, di-, tri-, tetra-, penta- and hexa-nucleotide sequences with a minimum repeat number of 12, 6, 5, 5, 4 and 4, respectively, were considered the search criteria. We scanned and counted the SSRs in all unigenes and the CDSs predicted in our study.

To mine SNP marker between *C. frutescens* and *C. annuum*, we realigned individual reads, which derived from xiaomila (*C. frutescens*), against *C. annuum* contigs (http://www.bioingenios.ira.cinvestav.mx:81/Joomla/) using SOAPaligner software (Release 2.21, 08-13-2009). The option of realignment was described previously. The thresholds for SNP identification were as follows: 1) the number of unique mapped and non-duplicate reads supporting a SNP had to be ≥4, 2) the consensus quality score had to be ≥20 (The quality score is a Phred score, generated by the program SOAPsnp, quality score 20 represents 99% accuracy of a base call), 3) the SNPs had to be at least 10 bp away from each other and 4) each haploid must have at less 10% approval rate of reads.

## Results

### RNA-Seq library sequencing and filter

Using Illumina sequencing, we generated 27,533,332 raw reads from a 200 bp insert library. After removal of low-quality reads (see Materials and Methods), a total of 26,405,238 high-quality PE reads and 535559 SE (single read of pair-end sequencing) were identified. These reads contained a total 2,339,147,147 nucleotides and 97.74% Q20 bases (base quality more than 20 and an error rate of less than 0.01), and remained for *de novo* assembly.

### Assessment of assembly program and parameters

Different software analysis will have great influence on the resulting transcriptome assembly. K-mer is one of the most important parameters in these assembly softwares used Bruijn graph algorithm. To obtain the optimal program and parameters for our transcriptome data, we used Trinity and Velvet-oases with different k-mer and analyzed each result, including total used reads, total length, N50 length, total contig (the number of contig, which at least 200 bp in length) and accuracy of assembly. We used the 25-mer in Trinity, which is recommended by its author [27]. Using this parameter, we found that total used reads, total length, N50 and accuracy are 85.74%, 41,646,449 bp, 1,108 bp and 98.31%, respectively (Table 1). In Velvet-oases, we chose 7 different k-mers (21, 25, 29, 31, 37, 41 and 47), and the results suggested that the k-mer length is related inversely to the total length, N50 length and number of contigs, which was similar to the reported results [35]. We found the best assembly result was 41-mer, as it had the highest total used reads and assembly accuracy (76.56% and 97.87%; Table 1). The total length, N50 length, total contig and accuracy were nearly comparable in both Velvet-oases and Trinity, but the total used reads in Trinity (85.74%) was about 9% more than that of Velvet-oases. Overall, Trinity is preferable to Velvet-oases in our study.

**Table 1:** Comparison of *de novo* assembly using Trinity and Velvet-oases programs.

	Trinity		Velvet						
k-mer	25	25#	21	25	29	31	37	41	47
Total used reads (%)	85.74	83.15	68.62	71.83	72.55	72.96	73.79	76.56	71.51
Total length(Mb)	41.65	38.51	67.75	60.52	54.32	51.42	44.10	39.95	34.12
N50(bp)	1,108	1,076	1,478	1,430	1,378	1,335	1,194	1,113	950
Total contig*	57,168	54,045	69,281	63,714	59,508	57,887	54,457	52,563	50,511
Accuracy (%)	98.31	99.55	94.65	96.11	97.16	97.39	97.91	97.87	97.36

* Represents the number of contigs that at less 200 bp in length.

# represents the result of TIGCL and Phrap for reduce the redundancy after Trinity with 25-mer assembly.

### The result of assembly

**Figure 1:**
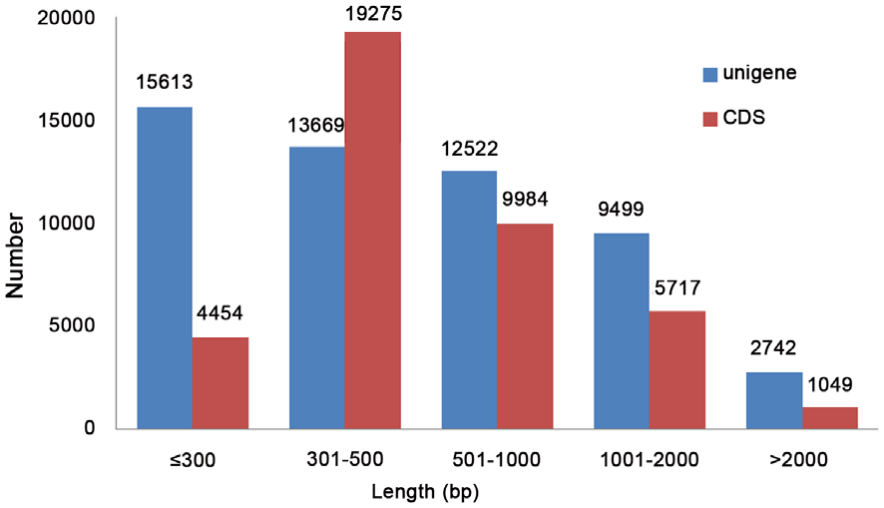
Size distribution of the unigenes and CDS. The blue and red bars indicate unigene and CDS, respectively.

We used Trinity with a 25-mer parameter to assemble our data (Table 1). Then, we used TGI and phrap to reduce the redundancy. Finally, we generated 54,045 non-redundant unigenes, comprising a total length of 38,505,452 bp (Text S1). The average length, N50 length, and average depth of these unigenes were 712 bp, 1,076 bp, and 60×, respectively (Table 1). This assembly produced a substantial number of large unigenes: 24,763 unigenes (45.82%) longer than 500 bp, 12,241 unigenes (22.65%) longer than 1,000 bp, and 2,742 unigenes (5.07%) longer than 2,000 bp (Figure 1).

To evaluate the accuracy of the assembled sequence, we realigned all the usable sequencing reads onto the unigenes. The reads coverage indicated that 95.50% of unigenes were covered full length by reads and the depth at least one read (high assembly accuracy; higher than Trinity only, 81.13%); 99.55% (higher than Trinity only, 98.31%) of unigenes were covered more than 90% length by reads. And approximately 83.15% (less than Trinity only, 85.74%) of the reads could be mapped on the unigenes. The total contig and total length was reduced by 3,123 and 3,140,997 bp, respectively (Table 1).

### Annotation

The unigenes were aligned to three public protein databases (Nr, Swiss-Prot and KEGG). A total 39,089 unigenes (72.33%) were annotated in these four databases. The number of homologous sequences in Nr was the most of all, followed by Swiss-Prot and KEGG (Table 2).

**Table 2:** The results of annotation on unigenes by different databases.

Database	number
Public database	39,089
Nr	38,927
SwissPort	23,514
KEGG	21,663
Potato	43,871
blastx^1^	38,867
blat^2^	33,260
Tomato	42,151
blastx^1^	39,431
blat^2^	29,066
EST database (*C.annuum*)	42,068
blastn	39,946
tblastx	39,907
**Total**	**50,075**

Note:

1 presents the unigenes annotated by whole protein sequences using blastx,

2 presents the unigenes annotated by whole genome using blat.

To identify the homologous genes in two related plant species, the unigenes were realigned to the tomato and potato genomes and their whole proteome. 43,871 unigenes realigned to the potato genome (33,260 using blat and 38,867 using blastx); and 42,151 unigenes realigned to tomato genome (29,066 using blat and 39,431 using blastx; Table 2). These data suggested that the chili pepper is more closely related to the potato than the tomato at the genetic level.

**Figure 2:**
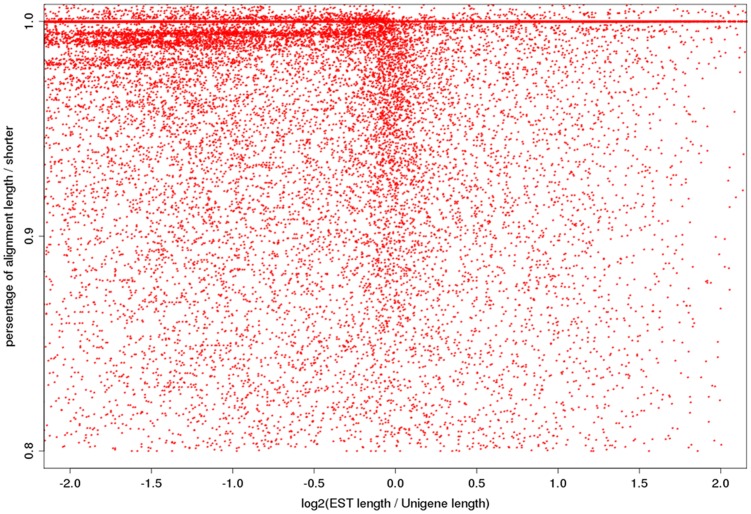
Illustrated the partly distribution (ratio of alignment/short no less than 0.8) of homologous length and aligned length. The X axis represents the ratio is length of pepper EST/unigene length, the Y axis is represents the ratio of alignment length/shorter between pepper EST and unigene.

To analyze of homologous was carried out against pepper (*C. annuum*) ESTs database using blastn and tblastx. A total 42,068 unigenes (77.84%) were searched homologous in ESTs database (Table 2). Then, we selected the best hit to each unigenes using blastn, and illustrated the partly distribution of homologous length and aligned length (Figure 2). This data showed that our result obtained longer transcripts than its homologies (840 bp and 541 bp respectively). Statistics suggestion that the average length and expression value (FPKM: Fragment Per Kilo base-pair per Million mapped fragments [37]) of unigenes, which has hit against the pepper (*C. annuum*) ESTs database, are 840 bp and 25.4 FPKM; and the remained unigenes are 351 bp and 6.9 FPKM, respectively.

A total 50,075 (92.65%) unigenes were aligned to homologous sequences in public databases, potato genome, tomato genome and pepper (*C. annuum*) ESTs database (Table 2). The remaining 3,970 (7.35%) unigenes may be the novel transcripts and special genes in to the *C. frutescens* chili pepper.

### Predicted CDS

Based on the three public protein databases, we got a total of 40,479 CDSs (38,319 CDSs predicted by blastx and 2,160 by ESTScan), among which 1,049 were over 2000 bp and 16,750 were over 500 bp (Figure 1). Unigenes not identified as coding sequences were likely either too short to reach the criterion of CDS prediction or were non-coding RNAs. The 15,613 unigenes less than 300 bp were not well annotated by public databases and identified the CDS. Putative non-coding RNAs need to be validated in the future study.

### The genes involved in capsaicinoids biosynthesis

In this study, we detected all (total 53) genes in Mazourek's pathway including 71 transcripts (Text S2). The expression level of almost all genes at less one transcript was over 20 FPKM, except six genes. Notably, the 3 enzymes Thr deaminase (TD), Dihydroxyacid dehydratase (DHAD) and Prephenate aminotransferase (PAT), for which candidate sequences have not been previously reported, were detected in our study (Table 3). TD and DHAD mainly participate in valine, leucine and isoleucine biosynthesis, as well as pantothenate and coa (Co-enzyme A) biosynthesis. PAT belongs to the family of transferases, specifically the transaminases, which transfer nitrogenous groups. This is the first report about them in chili pepper.

**Table 3:** Identity new transcripts (genes) on the capsaicinoids biosynthetic pathway.

Name	Abbreviation	Most Similar Arabidopsis Locus	Percent Identity	Unigene of chili pepper
Dihydroxyacid dehydratase	DHAD	AT3G23940	74.04	CL3547.Contig1_peper
Thr deaminase	TD	AT3G10050	72.41	CL3363.Contig1_peper
			66.37	CL3363.Contig2_peper
Prephenate aminotransferase	PAT	HM638413	70.03	Unigene4452_peper

In addition, the phenylpropanoid and benzenoid metabolisms and the medium-length, branched-chain fatty acid biosynthesis were classically considered as parts of capsaicinoid biosynthesis, and further included Phe and branched-chain amino acid biosynthesis [36], [17]. In our study, it is the necessary prerequisite to choice of the candidate genes that the expression value must over 20 FPKM. There were 197 transcripts encoding putative metabolic enzymes of these 5 pathways, including 35 also found in the Mazourek's pathway, leaving 162 as new candidate genes predicted by the KEGG analysis.

**Figure 3:**
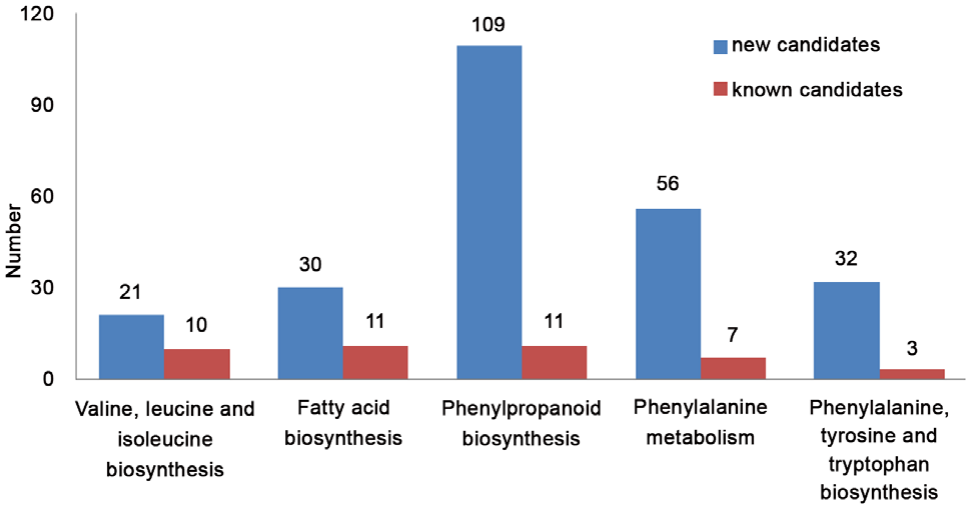
The candidates on the relative capsaicinoids biosynthetic pathway. The blue and red bars represent new candidates and known candidates from Mazourek, 2009.

Of the new candidates, including the same genes in different pathways, there were 21 genes in the pathway of valine, leucine and isoleucine biosynthesis, 30 genes in the pathway of fatty acid biosynthesis, 109 genes in the pathway of phenylpropanoid biosynthesis, 56 genes in the pathway of phenylalanine metabolism, and 32 genes in the pathway of phenylalanine, tyrosine and tryptophan biosynthesis (Figure 3).

### Putative Molecular Markers

**Figure 4:**
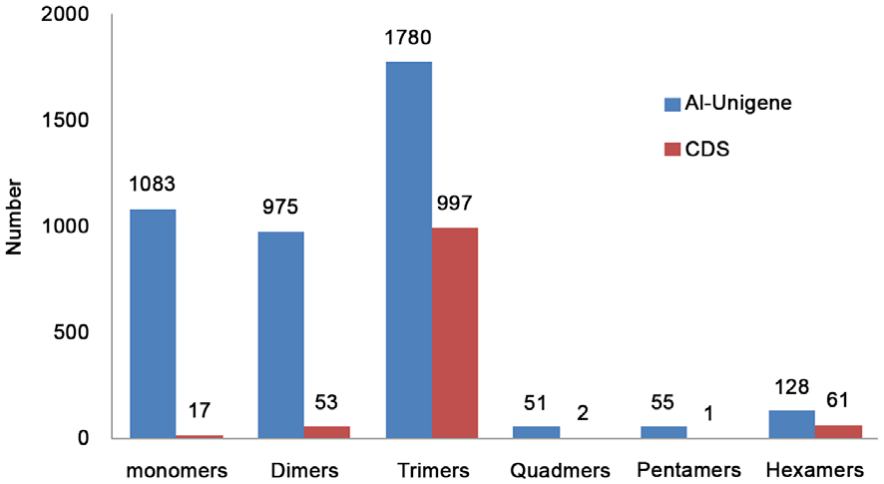
The number of SSR in all unigenes and CDS. The blue bar represents SSR markers in all unigenes, and the red bar represents SSR markers in CDS.

In this study, all 54,045 unigenes were used to detect SSRs, and a total of 4,072 SSRs were identified in 3,607 (6.7%) unigenes using MISA. Of these unigenes, 408 unigenes contained more than one SSR. The tri-nucleotide SSRs represented the largest fraction (43.7%), followed by mono-nucleotide (26.6%) and di-nucleotide (23.9%) SSRs (Figure 4 and Text S3). Interestingly, 1,131 out of the 3,607 SSRs were derived from CDS, and 1,058 (93.5%) of these CDS-SSRs were tri- and hexa-nucleotide motifs (Figure 4). These results suggested that tri-nucleotide (or a multiple of three) insertion or deletion would be the most popular type in CDS, as they seldomly generated frameshift mutations or termination codons. This phenomenon corresponds to natural selection. Additionally, a total of 9,150 putative SNPs, which was detected between *C. frutescens* and *C. annuum*, existed in 3,349 contigs, in which 5,457 were transitions and 3,693 were transversions (Table 4 and Text S4).

**Table 4:** SNP statistical information based on mapping *C. frutescens* reads in reference to *C. annuum* contigs.

SNP-type	Number
Transversion	3,693
C-G	782
A-T	1,017
A-C	925
G-T	969
Transition	5,457
A-G	2,750
C-T	2,707
**Total**	**9,150**

## Discussion

### The impact of different platforms of transcriptome sequencing

The transcriptome sequencing enables various functional genomics studies for an organism,especially for many plants and insects with complex genomes. The NGS (Next Generation Sequencing) technologies provide a low cost and rapid method for transcriptome sequencing and characterization. Roche's 454 GS FLX and Illumina/Solexa Genome Analyzer have been mainly used in *de novo* transcriptome assembly [38]–[43]. Although Roche's sequencing technology can produce longer reads, Illumina platform can obtain deeper coverage and higher accuracy than 454 technology with the same cost, which is beneficial for gene discovery [43]. In our results, the unigenes derived from our data, which were sequenced by Illumina system, are longer than its homologies from the pepper (*C. annuum*) ESTs database, which were sequenced by Roche's 454 and Sanger system (840 bp and 541 bp respectively; Figure 2). Furthermore, statistics suggest that the unigenes without hit against the pepper (*C. annuum*) ESTs database possess the characteristic of shorter length and lower expression value (840 bp and 25.4 FPKM; 351 bp and 6.9 FPKM, respectively).

### Assembly quality of unigenes

For most NGS data sets, low quality scores indicate possible sequencing errors, which would affect the assembly. There are different ways to deal with the errors, such as removal, trimming or correction to improve the assembly quality and decrease the amount of random access memory (RAM) required [35], [44]–[47]. However, k-mer-based error correction carries a side effect, in that reads derived from rare transcripts may also be removed [44]. In order to obtain high-quality reads, we used a trimming strategy for filtering the reads in our research, and the Q20% is 97.74%.

The short read length needs efficient assembly software to reconstruct all of the transcripts and their variants. There are two challenges: 1) the sequencing depth of transcripts can vary by several orders of magnitude, so it is difficult to balance sensitivity to low-abundance transcripts and the error of assembly. 2) Transcript variants from the same gene can share exons and are difficult to resolve unambiguously [44]. Recently, Garg [35] reported that Velvet-oases was better than ABySS, CLC Genomics workbench and SOAPdenovo. Additionally, Grabherr [27] reported Trinity was a new special software for *de novo* transcriptome assembly, and the number of Trinity-assembled transcripts was substantially higher than achieved by other *de novo* assemblers, such as TransABySS, ABySS, and SOAPdenovo. In our research, we assessed efficiency of assembly between Trinity and Velvet-oases, and our result showed that Trinity obtained better total used reads (Trinity was 85.74%; Velvet-oase was 76.56%) and assembly accuracy(Trinity was 98.31%; Velvet-oase was 97.87%) than Velvet-oases, while the other results were comparable (Table 1). These data suggested that Trinity could reconstruct more transcripts and their variants with high assembly accuracy. Furthermore, Trinity can obtain substantially polymorphic transcripts which were identified by a small variation (a number of SNPs or small Indels) [27]. It was useful to detect the differences in detail between alleles. In conclusion, there are two important factors for *de novo* transcriptome assembly: 1) the performance of software and 2) the quality of reads.

To reduce the redundancy, we used the TGI Clustering Tool followed by Phrap assembler to obtain distinct sequences. And the result showed that the total contig and total length was reduced by 3,123 and 3,140,997 bp, respectively, and the high assembly accuracy unigene was up to 95.50% (higher than Trinity only, 81.13%). These data showed that this strategy was very effective to reduce the redundancy. We generated a total of 54,045 high-quality unigenes of chili pepper, and these data will provide new insights into the biology of *Capsicum frutescens* (Table 1 and Figure 1). These strategies of *de novo* transcriptome assembly can be helpful for other similar studies.

### The genes involved in capsaicinoids biosynthesis

Capsaicinoids have been widely used for food, medical, and pharmaceutical purposes [2]–[8]. The general capsaicinoid biosynthetic pathway was established at the end of the 1960s [11]. Until 2009, a preliminary model for capsaicinoid biosynthesis was built by Mazourek [17], and most of the candidates were cloned, except 3 structural genes. In this study, we first identified the 3 genes, TD, DHAD and PAT, by transcriptome sequencing, in addition to other genes in this pathway. Uncovering the 3 specific functional genes was easier with the utility of transcriptome sequencing than traditional methods. Furthermore, almost all the genes had a high expression of the FPKM over 20 (the largest one was 370.1), and this suggested they had an important function at this stage, from 20 to 30 DAF, in which, capsaicinoids are synthesized actively and accumulate in chili pepper [24], [48]. So far, the genetic information of the capsaicinoid biosynthetic pathway predicted by Mazourek has been completed. Nonetheless, Mazourek's model was just based on homology relationships, as opposed to the discovery of novel or regulatory genes [17]. Then, we used KEGG to predict the other 197 transcripts in 5 pathways, which were related to capsaicinoid biosynthesis (Figure 3). These may be new candidates involved in capsaicinoid biosynthesis. The roles of these transcripts in capsaicinoid biosynthesis will be validated in the future.

### Putative molecular marker

The transcript-based markers are an important resource for determining functional genetic variation [49]. Among the various molecular markers, simple sequence repeats (SSRs) are highly polymorphic, easier to develop, and serve as a rich resource of diversity [35]. Detection of mutants by comparative SNP analysis in non-pungent and pungent chili peppers might render new information on structural or regulatory genes that participate in capsaicinoid biosynthesis [11]. And there are some other SNP and SSR research reports in *C. annuum* [21], [32]–[33], [50]–[53]. But the SNP markers between *C. frutescens* and *C. annuum* have not yet been reported. In this study, we predicted a total 9,150 SNPs and 4,072 SSRs molecular markers (Table 4, Figure 4 and Text S2). These SNP markers are represent the partly difference between *C. frutescens* and *C. annuum*. It could lay a platform for better understanding the polymorphisms of chili pepper. However, all the predicted molecular markers need to be validated to rule out false positives and sequencing errors. Our result can provide substantial molecular markers for the study of the chili pepper.

### Conclusions

This study was aimed to obtain fundamental molecular knowledge of capsaicinoids, which are produced in chili pepper. Some noteworthy results presented in this study are: 1) a significant strategy of *de novo* transcriptome assembly can be potentially used for any species; 2) a significant number of putative genes which are related to the pathway of capsaicinoid biosynthesis are identified within the derived sequences; and 3) a number of SSR and SNP markers are predicted, which upon validation could facilitate the identification of polymorphisms within chili pepper populations. Although the molecular functions of capsaicinoid biosynthesis genes remain unknown largely, the present transcriptome analysis provides valuable information regarding the biosynthesis of capsaicinoids. Also these characteristic sequences will provide new insights into the biology of *Capsicum* spp.

## Supporting Information

Text S1 **The unigene sequence.**
(RAR)

Text S2 **The information of the transcripts on the capsaicinoids biosynthetic pathway.**
(RAR)

Text S3 **The information of SSR derived from all unigene.**
(RAR)

Text S4 **SNP information based on mapping** ***C. frutescens*** **reads in reference to** ***C. annuum*** **contigs.**
(RAR)
